# Contraceptive counselling and uptake of contraception among women with cardiovascular diseases: a systematic review and meta-analysis

**DOI:** 10.1007/s00392-024-02472-9

**Published:** 2024-07-10

**Authors:** Tesfaye Regassa Feyissa, Shahinoor Akter, Melissa L. Harris

**Affiliations:** 1 Deakin Rural Health, School of Medicine, Deakin University, Warrnambool, Australia; 2 Geohealth Laboratory, Dasman Diabetes Institute, 15462 Kuwait City, Kuwait; 3 John Richards Centre for Rural Ageing Research, La Trobe Rural Health School, La Trobe University, Albury-Wodonga, Victoria Australia; 4 Centre for Women’s Health Research, College of Health, Medicine and Wellbeing, The University of Newcastle, Newcastle, New South Wales Australia; 5 Hunter Medical Research Institute, Newcastle, New South Wales Australia

**Keywords:** Cardiovascular, Heart, Contraception, Contraceptive counselling, Women, Interventions

## Abstract

To address the lack of clarity regarding contraceptive counselling and uptake of contraception among women with cardiovascular disease (CVD), this study aimed to conduct a systematic review and meta-analysis on contraceptive counselling and the subsequent uptake of contraception among women with CVD. A search across six databases identified 1228 articles, with 11 studies (2580 participants) included. The pooled prevalence of contraceptive counselling was 63%, varying from 36 to 94% in individual studies. Inconsistent delivery and documentation of counselling were noted, along with a lack of knowledge about pregnancy complications and misconceptions regarding CVD severity. For contraceptive uptake (*n* = 5), the pooled prevalence was 64% (95% confidence interval, 45 to 82%). Women with CVD were also found to use less effective methods as well as methods not recommended for their condition (e.g., combined hormonal methods among participants with absolute or relative contraindications). Improving contraceptive counselling and choices for women with CVD can not only enhance knowledge and decision-making for women with CVD in line with their reproductive goals and preferences but reduce high-risk unintended pregnancies and adverse pregnancy outcomes.

**Graphical Abstract:**

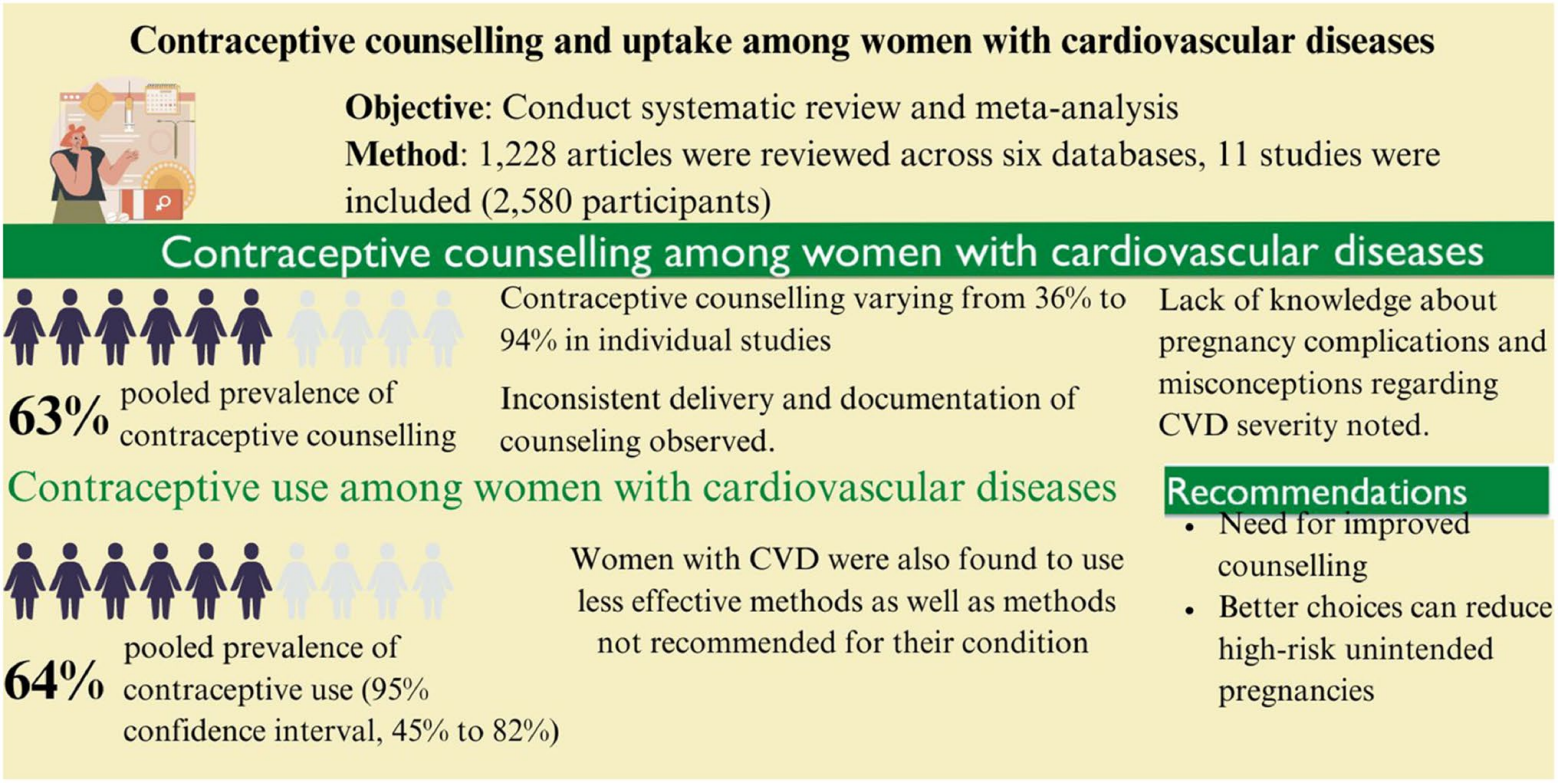

**Supplementary Information:**

The online version contains supplementary material available at 10.1007/s00392-024-02472-9.

## Introduction

Cardiovascular disease (CVD) is the leading cause of morbidity and mortality in women worldwide [1], with an estimated 275 million diagnosed cases in 2019 [2]. The prevalence of CVD among women has increased significantly over the past decade and is occurring earlier in the lifespan [2, 3]. As such, up to one-third of cases are being diagnosed during women’s reproductive years [4]. Particularly, among women aged 20 to 29 years, the prevalence of CVD (e.g., congenital heart disease and acquired disease such as coronary heart disease, heart failure, stroke, and hypertension) has been estimated at 11.5% [5]. Reproductive-aged women with CVD have a higher risk of adverse maternal and infant outcomes (e.g., small for gestational age and preterm delivery) [6, 7]. Pregnant women with CVD also have a higher risk of fetal morbidity, including growth restriction and premature delivery [8].

Adverse maternal and fetal outcomes among women with CVD can be significantly reduced by preventing high risk unintended pregnancies [9–12]. Organisations such as the American College of Cardiology and the Cardiac Society of Australia and New Zealand guidelines recommend that clinicians assess the family planning needs of women with CVD and ensure women who do not wish to become pregnant are protected and those who do wish to become pregnant to be able to plan their pregnancies appropriately for their specific disease and severity [9, 13, 14]. However, many women with CVD face an increased risk of unintended pregnancies due to non-use of contraception or use low efficacy methods [12, 14, 15]. The contraceptive needs of women with cardiac disease are especially challenging to navigate due to the variability in potential risks associated with both contraception type and the nature and severity of the cardiac disease [16]. Accurate and comprehensive contraceptive counselling can help women understand and consider CVD risks, make informed decisions about contraception and family planning [4], and reduce the risk of unintended pregnancy in high-risk patients [14].

Importantly, the UK medical eligibility criteria provide a guide for healthcare providers to assist women with chronic diseases such as CVD with their contraceptive decision-making. Women with CVD have several contraceptive options available to them to use, with some exceptions [14, 17]. Long-acting reversible contraception (LARCs), like intrauterine devices (IUDs) and implants, preferred for their high efficacy and safety, are particularly recommended for women with or at risk of CVD. However, women with certain conditions may be advised to avoid oestogen-containing methods [14, 18]. For example, the combined oral contraceptive pill can increase the risk of blood clots and high blood pressure, so it is recommended to initiate contraceptive discussions more frequently and provide improved and adequate contraceptive counselling for women with complex heart disease [19, 20]. As a standard practice, contraceptive counselling should encompass information about highly effective and long-term safe options like LARCs while being tailored to individual reproductive goals and health conditions (Table 1). While providing contraceptive counselling to women with CVD is crucial, it is not entirely clear how current contraceptive counselling is provided. This study therefore aimed to systematically review the available evidence and produce an overall summary estimate of contraceptive counselling and uptake of contraception among women with CVD.

**Table 1:**
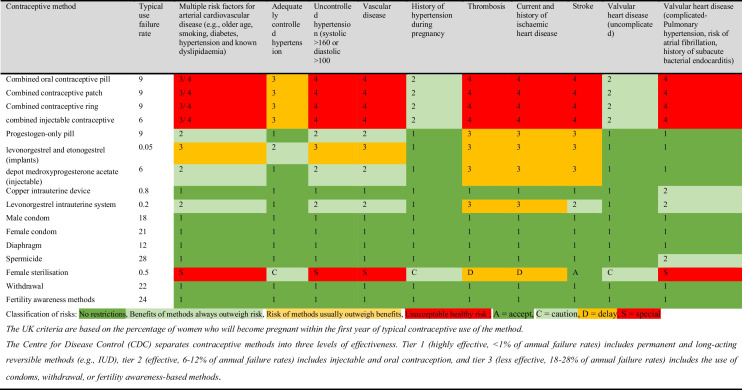
UK medical eligibility criteria for contraceptive use [21] and method effectiveness

## Methods

### Protocol and registration

This systematic review adhered to the 2020 Preferred Reporting Items for Systematic Reviews and Meta-Analyses (PRISMA) guidelines [22]. The protocol for the review was registered with PROSPERO (number CRD42021287927).

### Eligibility criteria

Studies were included if they reported on the provision of contraceptive counselling for women of reproductive age (15 years and above) with a history of CVD. Studies included both congenital and acquired forms of CVD (including hypertension). Peer-reviewed original research involving quantitative, qualitative, and mixed methods published in the previous 10 years was considered. Ten years was deemed appropriate to ensure that the reported evidence and practice on contraceptive counselling for women with CVD was current to allow the making of relevant evidence-based recommendations. Studies were excluded if contraceptive counselling for women with CVD could not be disaggregated. Randomised controlled trials on CVD treatment as well as case studies, editorials, opinion pieces, research letters, and literature reviews were also excluded, although the reference lists were manually searched to ensure no relevant articles were missed.

### Data sources and search strategy

Data were identified by searches of six electronic databases: MEDLINE (Ovid), EMBASE (Ovid), CINAHL (EBSCO*host*), MIDRIS (Ovid), and PsycInfo (Ovid). Where possible, search terms were matched to MeSH or database specific subject headings, using keyword indicators. An initial search strategy covering the concepts of cardiovascular disease (e.g., heart or cardiac or myocardial diseases) and contraceptive counselling or family planning services was developed by TRF and tested in MEDLINE using Boolean operators (AND/OR). The search strategy was then refined in consultation with MLH and the College of Health, Medicine, and Wellbeing Librarian. Initial searches were performed in November 2020 and were updated in March 2023. The searches were limited to recent articles published between 2012 and 2023, written in English and involving human subjects (where databases allowed such limitations). This is particularly important given the increase in knowledge and acceptability of LARC for young women as well as recognition of medical eligibility guidelines for their use in women with chronic disease [23]. The final full search strategy is shown in Supplementary File 1.

### Study selection

**Fig. 1:**
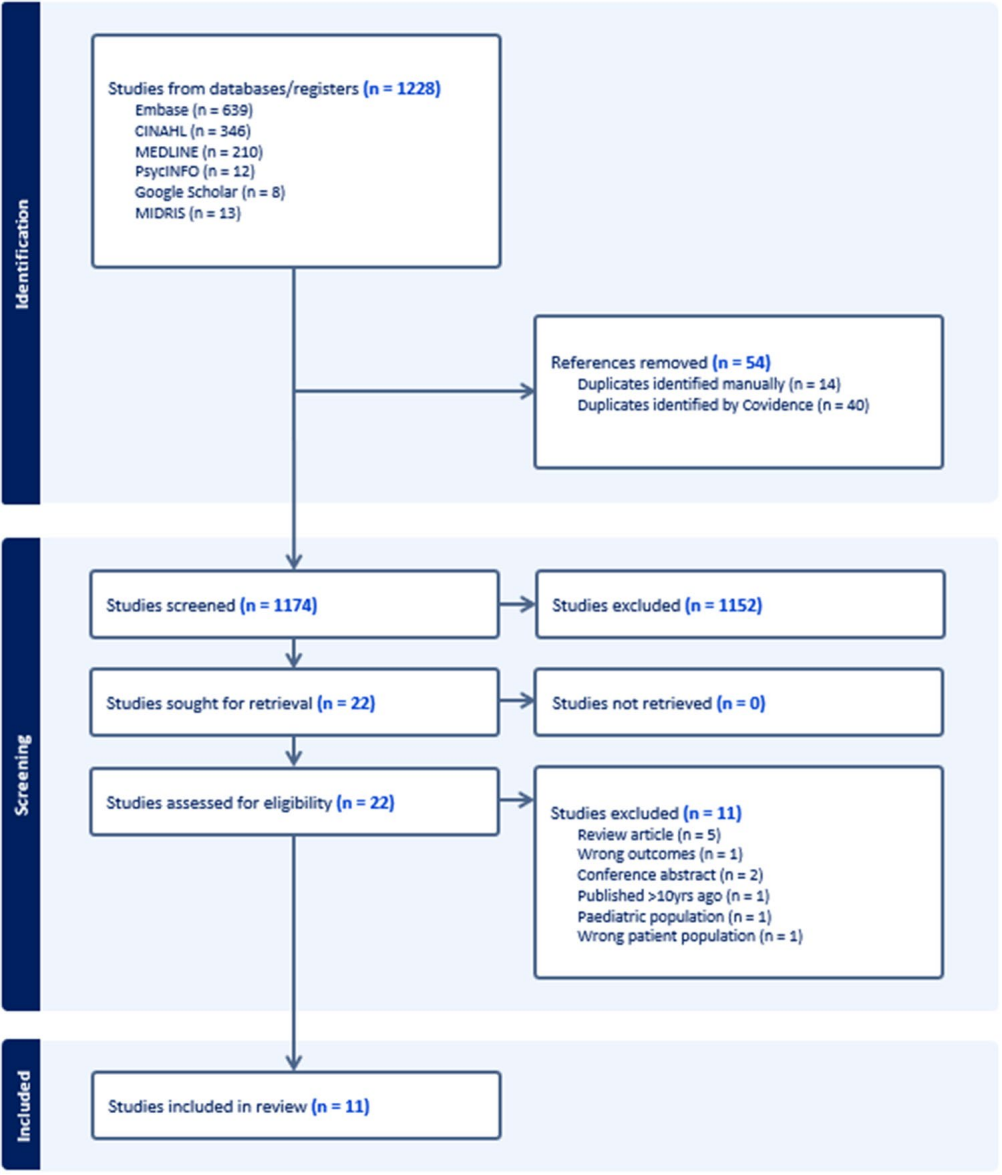
PRISMA study selection flow diagram

Articles were downloaded and exported into the systematic review management program, Covidence (Veritas Health Innovation, Melbourne, Australia), and duplicates were removed. Articles were then screened for eligibility based on title and abstract (Fig. 1). Abstracts were independently screened by two researchers (TRF and MLH), and discrepancies were resolved by consensus. Studies that met the eligibility criteria were retained for full-text review (TRF and SA). If a peer-reviewed journal abstract was eligible, the whole article was read for inclusion. Any disagreements were resolved through consensus. For this review, two articles were resolved through the involvement of a third reviewer (MLH).

### Data extraction and analysis

Two researchers (TRF and MLH) extracted data from each study independently. The data extraction sheet contained the first author, publication year, country, aim, design, and study characteristics such as participant description (including type of CVD), sample size, study time frames, key findings, and limitations (Table 2). The results were summarised narratively. This was followed by a summary prevalence estimate of contraceptive counselling and subsequent uptake of contraception among women with CVD which was calculated by pooling the study-specific estimates. Random effects meta-analysis that considered heterogeneity between studies was used for pooling. The *Q* test and the *I*^2^ statistic were used to assess the heterogeneity between studies using Stata 17. A score of > 75% was used to indicate heterogeneity between studies. Subgroup analysis by study design was also conducted to investigate the sources of heterogeneity for the prevalence of contraceptive counselling.

**Table 2:** Characteristics of included studies included in this review

Author and year	Country	Design and setting	Participant characteristics	Data collections	Contraceptive counselling	Contraceptive use (prevalence, facilitators, and barriers)
Cauldwell 2017 [24]	UK	Retrospective cohort study; Royal Brompton and Chelsea and Westminster Hospitals	76 women classed as mWHO 3 and 4 heart disease who had a pregnancy from at least 20 weeks gestation. Of the 76 women in the study, 65 had an established cardiac diagnosis prior to their first recorded pregnancy	Cardiac disease and pregnancy database	Of the established cardiac disease women, 63% attended PCC. Of the 14 that did not attend PCC, 5 referred to our service from another unit without PCC, and 2 previously defaulted from follow-up. There was no evidence that the remaining 7 cases were ever offered PCC. Out of 102 pregnancies, 63 had documented PCC. Patients who were referred and seen in PCC were statistically more likely to be older at first delivery than those who did not have PCC (mean age 30 years (SD4.7 years) vs. mean age 26 years (SD 6.1) *p* = 0.006) *Source*: combined ACHD cardiologist and obstetrician and follow-up from cardiology team	40 pregnancies were unintended. There were 5 pre-viable terminations of pregnancy between 20 and 22 weeks, all for medical reasons. There were corresponding associations with maternal death (*p* = 0.002): 3% in the low-risk, 3% in the intermediate risk, and 33% in the high-risk group died.
Hinze 2013 [25]	USA	Cross-sectional survey; single tertiary adult CHD clinic	83 women aged 19 or older women with CHD	Survey	*Contraceptive counselling rate*: 36% *Contraceptive counselling* that addressed the heart condition—65% reported that a healthcare professional had never discussed their options for birth control with respect to their heart condition. Of the 38 participants who reported receiving contraceptive counselling that addressed their heart condition, only 47% received such counselling before their first engagement in intercourse. 64% of women said that they had not received both contraceptive and reproductive counselling *Source*: Heart-specific counselling—a paediatric cardiologist was involved with 42% of their first contraceptive counselling and 13% for their most recent counselling. A cardiac specialist (paediatric cardiologist, adult cardiologist, or cardiothoracic surgeon) provided contraceptive counselling at least once for 57% overall, only 25% of all study participants received heart-specific contraceptive counselling from a cardiac specialist. Others reported that heart-specific counselling first came from a primary care physician (26%) or an obstetrician/gynaecologist (24%). Most recently, 29% received counselling from a primary care physician and 24% from an obstetrician/gynaecologist *General contraceptive counselling*—Participants reported receiving counselling that did not include a discussion of their heart condition most frequently from a family medicine physician (73%) or an obstetrician/gynaecologist (55%). This group included 40 participants (48%) *Facilitators*: older women were more likely to report receiving such counselling (*P* = .04) *Higher pregnancy WHO class—*Women in higher pregnancy WHO classes were significantly more likely to have received counselling than those in lower WHO classes (*P* < .002)	Of the 45 participants who reported never receiving contraceptive counselling that addressed their heart condition, 7% were found to have a history of using a method that was contraindicated given their underlying heart condition. Conversely, of the 38 participants that reported receiving contraceptive counselling that did address their heart condition, a twofold greater percentage (*n* = 7, 18%) was found to have used a contraindicated type; however, this was not significant (*P* = .17). The 40 participants who received counselling that did not include a discussion of method appropriateness given their heart condition did not tend to use a contraindicated contraceptive method any more frequently than the 30 women who never received such counselling (*n* = 4 vs. 5) (*P* = .48). Of these 40, three-fourths (*n* = 30, 75%) reported that they used the method recommended to them, whereas7 (18%) did not use that method, and 3 (7%) did not remember. Of the 30 women that reported using the recommended method(s), 3 (10%) were found to have used methods that were contraindicated The combined hormonal oral contraceptive pill was the method most frequently used in the last 3 months (34%), with an additional (40%) reporting history of use at a point in time not occurring in the previous 3 months. The non-permanent methods least likely to have ever been used include the progesterone implant (*n* = 81, 98%), the copper IUD (*n* = 80, 96%), the combined estrogen/progesterone transdermal patch (*n* = 80, 96%), the progesterone-only mini-pill (*n* = 76, 92%), and progesterone-only IUD (*n* = 76, 92%). Over half of the women (*n* = 52, 63%) did not know if there was a method or methods, they should not use given their heart condition. Eight of the women were CHC class 4, and an additional 8 were CHC class 3, for a total of 16 women at high risk of adverse events occurring secondary to the use of CHC.
Kaemmerer 2012 [26]	Germany	Clinical assessment and cross-sectional survey; 2 tertiary care centres for adults with CHD	536 confirmed CHD with median of 29 years; the underlying congenital anomaly was identified as simple in 127 cases (23.7%), as moderate in 275 cases (51.3%), and as severe in 134 (25.0%)	Self-administered cross-sectional survey by women with CHD and their treating physicians with linked medical and surgical records	*Prevalence*: 85% received contraception information from physicians; 45.9% from friends; 28.3% from parents; 24.7% from the internet; 23.2% from newspapers; and 5.4% from others with the disease. 80.5% of women aged 18–24 years and 86.7% of those aged 25–34 were most likely to receive contraceptive information from a physician, followed by friends (59.2% and 43.6%, respectively) *Pregnancy counselling*: 83.8% from physicians; 49.9% from friends; 45.8% from parents; 30.2% from the internet; 28.8% from newspapers etc.; and 8.9% from others with the disease. When looking across age groups, those aged 18–24 years received 75.5% information from a physician and 59.8% from parents; for those aged 25–34 years, 86.5% received information from physicians and 52.8% from friends Low rating regarding information reported on contraception (effects and side effects) across all age groups and low score for risk of pregnancy in 18–24 years and > 45 years *Source*: For contraceptive counselling, patients of all age groups primarily communicated with a physician. The number of women aged 35 years or older who had sought to receive information from their parents was small. In all age groups, few women received information from other affected patients *Facilitators*: There was a slight difference between different age groups. The lowest grade of contentedness was encountered regarding sexuality and the side effects and benefits of contraceptive options in women aged 45 years or older *Sources*: Physician was a key contact person; internet use was popular among those 18–34 years old	
Koerten 2016 [27]	Germany, Hungary, and Japan	A multicentre cross-sectional approach	634 women with CHD, included over a period of 12 months Of these, 45.7% (*n* = 290) indicated to be poorly informed, 30.6% (*n* = 194) felt moderately informed, and only 17.8% (*n* = 113) assessed themselves as being informed very well	Survey	How they felt informed about the impact of different contraceptive methods on CHD were answered by 597 of the surveyed women (94.2%)	Prevalence 58.0% (*n* = 368). Overall, the condom was used the most (38%), followed by oral contraceptives (30%) and coitus interruptus (11%) In all three risk classes (for maternal cardiovascular complications during pregnancy), the proportion of women currently using and having used exclusively contraceptive methods rated as “safe” was approximately equal (low risk: 72.1%, medium risk: 69.5%, high risk: 71.1%)
Lindley 2015 [28]	USA	Cross-sectional study; one university clinic	100 women with congenital heart diseases (CHD); aged 18–45 years (mean = 20.5 years)	Self-administered survey	*Prevalence*: 92.9% recalled ever discussing contraception with an obstetrician and gynaecologist, but only 45.9% recalled ever discussing these topics with a cardiologist 51.0% accurately assessed their cardiovascular pregnancy risk *Sources*: Obstetrician and gynaecologist; cardiologist	*Prevalence*: Of the sexually active women (83), 76% of the women were using any contraceptive. 36% of women were using tier I methods of contraception, 24.1% were using tier II methods, and 16% of sexually active women reported using tier III methods. The use of long-acting reversible contraceptives (LARC) was lower among the participants; only 11% used LARC.
Londono-Obregon 2017 [29]	USA	Cross-sectional survey; outpatient clinic in a large paediatric hospital	100; 54 females; patients with CHD aged 18 years and older; 70% of women were sexually active	Survey	*Prevalence*: 29% received contraceptive counselling. Of the 54 women, 25 (46%) identified their contraceptive options correctly; 42 (78%) women were classified as being at significantly increased risk for an adverse outcome during pregnancy, and of these, 20 (48%) identified this risk correctly. Of all patients surveyed, 72% did not know that having CHD placed them at increased risk for having a child with CHD *Source*: 54% of the females reported previous counselling on contraception by their cardiologists, primary-care doctors (26%), gynaecologists (32%), friends (25%), family members (46%), and schoolteachers (30%)	
Mechal 2022 [30]	Ethiopia	Cross-sectional study, cardiac clinic	284 reproductive-aged women with CVD: hypertension 90 (31.7%), chronic rheumatic valve disease 89 (31.3%), and congenital heart disease 47 (16.4%) Most of the study subjects were between the age of 19 and 34 years, with a mean age of 34 ± 7.4 years	Exit interview using a structured and pretested questionnaire	Prevalence: 117/284 (41.2%) were counselled on contraceptive utilisation; only 25% were linked with the family planning unit of the hospital	Prevalence: Contraceptive prevalence was 30.2% (*n* = 86/284). Overall unmet need for contraception was 36.0%. The most common reasons for the non-use of a contraceptive method were fear of drug side effects and drug interaction. Unmet need for contraception was found to be more likely among those who have not been counselled on contraceptive utilisation (AOR 6.7, CI 1.8–24.7) and those who lack partner support on contraception use (AOR = 6.2, CI: 1.91–19.8). Unmet need was also found to be more likely among women who have—never used contraception before (AOR = 3.2, CI 1.12–8.92).
Miner 2017 [31]	North America	Cross-sectional; 9 ACHD centres throughout North America	505 women > 18 years of age with CHD	48-item questionnaire regarding contraceptive use and perceptions of contraceptive counselling, and a medical record review	*Prevalence*: 84% reported receiving some form of contraceptive counselling but only 43% reported receiving counselling by ACHD provider *Source*: Only 43% indicated receiving counselling from their ACHD provider (physician, nurse practitioner, or physician assistant); 55% from gynaecologists; 11% from primary care physicians *Facilitators*: Hispanic women were less likely to report receiving such information compared to non-Hispanic women (69% vs. 89%, *p* = 0.001), as were older women compared to younger women (39.0 vs. 34.8, *p* = 0.027). Patients with CHD of great complexity were more likely to have documentation of contraceptive counselling than patients with less complex CHD (56.0% vs. 45%, *p* = 0.036) *Barriers*: Patients with CHD of great complexity were more likely to have documentation of contraceptive counselling than patients with less complex CHD (56.0% vs. 45%, *p* = 0.036)	The majority (86%) of the cohort had utilised contraception *Types*: barrier methods (87%), oral contraception (OC) 84%, intrauterine device (18%), Depo-Provera (15%), vaginal ring (7%), patch (6%), hormonal implant (2%), Plan B (19%), and sterilisation (16%)
Prabhakar 2021 [32]	UK	Retrospective comparative audit; single centre, tertiary maternity unit	113 reproductive-aged women 18–43 years *Types*: 45% CHD, 18% arrhythmias, 16% aortopathy, 11% cardiomyopathy, 7% rheumatic valvular disease, 3% miscellaneous *Treatment:* Prior to pregnancy, 25% were taking beta-blockers and 7% angiotensin inhibitors: 2 patients were on both beta-blockers and angiotensin inhibitors; 9% were anti-coagulated *Modified World Health Organization (mWHO) classification*: 50% stage I, 22% stage II, 14% stage II–III, 14% stage III. No patients were classified as WHO IV **Group 2**: Forty (60%) had a diagnosis of CHD, 15% arrhythmias, 5% aortopathy, 7% cardiomyopathy, 7% rheumatic valvular disease, and 6% miscellaneous. Prior to pregnancy, 22% were taking beta-blockers and 12% were anti-coagulated *mWHO Classification*: 33% stage I, 19% stage II, 31% stage II–III, 14% stage III, 3% stage IV	Electronic patient records	In Group 1, 45% of patients with a cardiac condition had counselling. Only 40% of those had documented maternal and fetal risks in their patient records. Women with CHD were more likely to receive counselling than those with alternative diagnoses. High-risk women were more likely to receive counselling than low-risk women In Group 2, 46% of patients received counselling. Of those, only 58% had documented risks. Women with CHD were more likely to receive counselling than those with alternative diagnoses. High-risk women were more likely to receive counselling than low-risk women In Group 1, risk documentation was not significantly different between high-risk and low-risk women. In Group 2, high-risk women had better risk documentation than low-risk women	
Rosman 2017 [33]	USA	Cross-sectional; registry including 27 states	177; 18 years and older, prior diagnosis of PPCM	Survey modelled after the Contraceptive Use Questionnaire from the Women’s Interview Study of Health	The healthcare team provided information regarding the potential risks of subsequent pregnancies to 75.1% of PPCM patients in this cohort. 3 out of 4 women reported that they discussed the importance of contraception to prevent PPCM recurrence with their healthcare team Source: healthcare team	Among contraceptive users in this PPCM cohort, 48.2% reported regular use of contraception to prevent future pregnancies, while 15.0% of women reported using contraception at least half of the time or most of the time. Tubal ligation (24.3%), condoms (22.0%), IUDs (16.4%) were the most common forms of contraception.
Sabanayagam 2017 [34]	USA	Cross-sectional survey; a single-site national adult CHD conference	77 women with any form of CHD aged ≥ 18 years, who had been pregnant at least once	50-question survey	*Prevalence*: 85% discussed contraception, 66% told they were at high risk for adverse cardiovascular events, whereas 20% reported that their cardiovascular risk during pregnancy was not discussed with them. Owing to their underlying cardiac condition, 30% of women were told that pregnancy was contraindicated. 29 with severely complex CHD:9 women were told that pregnancy was contraindicated, 7 were advised that they were at low risk, 8 did not know their risk assessment, and 5 did not answer the question. In the moderately complex group (*n* = 39), 12 were told that pregnancy was contraindicated, 15 were deemed to be at low risk, 5 did not know their risk assessment, and 7 did not answer the questions. None of the women with mildly complex CHD were told that pregnancy was contra-indicated. Out of the 13 women with single-ventricle physiology, 7 were told that pregnancy was contraindicated, 3 women did not know their risk assessment, 2 did not answer the question, whereas 1 woman thought that risk for adverse events was negligible The risk of cardiac complications during pregnancy was reviewed by their cardiologist in only 66% of the respondents. Fifty-two percent thought they could get pregnant regardless of their condition, whereas 11% were not sure despite having sought regular cardiology care. Out of all the women (*n* = 29) with conotruncal abnormalities including D-transposition of the great arteries, congenitally corrected transposition of the great arteries, double-outlet right ventricle, tetralogy of Fallot, and truncus arteriosus, only16 women had discussions regarding recurrence risk in their offspring. In women with congenital aortic stenosis/bicuspid valve (*n* = 6), only 4 had discussed recurrence risk with their cardiology providers *Sources*: cardiologist	Facilitators: Disease severity—the predominant groups among those women who were told they were at high risk for adverse cardiovascular events were those with single-ventricle physiology (*n* = 10) and tetralogy of Fallot (*n* = 9), including two women with concomitant pulmonary atresia.

### Methodological quality appraisal

The Joanna Briggs Institute (JBI) quality appraisal tools [35] were used to assess the quality of included studies. Two reviewers independently undertook the assessment. The reviewers independently rated each of the nine items (for cross-sectional studies) and eleven items (for retrospective cohort studies) as either “yes”, “no”, or “unclear”. Any disagreements were discussed and resolved by the reviewers. A higher score indicated a lower risk of bias, and the studies were categorised as having high risk (scores 1–3), medium risk (scores 4–6), or low risk (7 or more) based on their bias assessment.

## Results

### Study characteristics

A total of 1228 articles were initially identified. Of these, 54 articles were duplicates, 1152 articles were excluded during the title and abstract screening process, and another 11 were excluded after a full-text review as they were ineligible. A total of 11 studies published between 2012 and 2022 (involving 2580 participants) across six countries were included in this review (Fig. 1). Of the studies included, nine were cross-sectional, and two were retrospective cohort studies. Seven studies were conducted in the USA (one of them conducted throughout North America), and two were from the UK. All eleven studies were conducted in facility-based settings (e.g., hospitals) [25–34, 36]. Participants in all studies were women aged 15 years and older (Table 2).

The risk of bias of studies was assessed by the JBI checklist quality appraisal tool. The quality appraisal of the included studies, including both cross-sectional and longitudinal designs, showed methodological strengths and weaknesses. Among the nine cross-sectional studies, variations were observed in appraisal scores. One study [26] received the highest methodological rigour with a score of 8. Two other studies [25, 28] had an appraisal of 6. However, five studies [29–31, 33, 34] received lower appraisals ranging from 3 to 5, suggesting limitations. In contrast, both retrospective longitudinal studies [32, 36] had similar appraisal scores of 6. Overall, the studies showed limitations in specific areas such as a lack of describing study subjects and settings [34], measuring exposure [27, 29–31, 33], identifying and dealing with confounding factors [25, 28, 29, 31, 33, 34], and reporting follow-up time and completeness [32, 36] (Supplementary Tables 1 and 2).

### Cardiovascular disease classification

Eleven studies reported on CVD classification either through medical classification or self-report. A German cross-sectional study at two tertiary care centres involving women with congenital health diseases (CHD) aged 18 years or older classified CVD severity using the American College of Cardiology (ACC) recommendations. Of these participants, 52% had moderate CHD, and 25% had severe CHD. However, the treating physician classified 41% of the women as class II and 8% as class III–IV [26]. A cross-sectional study from the USA reported CVD classification based solely on self-report. Of the 77 women, 38% reported severely complex CHD, and 51% reported moderately complex CHD [34].

Five studies included disease severity as part of CVD classification [24, 25, 29, 31, 33], with three using the modified World Health Organization (mWHO) classification [24, 25, 29]. Using mWHO, a study from the UK reported a significant percentage of women with CVD were at moderate level [24]. A cross-sectional survey of nine Adult Congenital Heart Disease (ACHD) centres throughout North America found that women with CHD (aged 18 years or older) primarily had moderate (45%) or great complexity (36%) [31]. Another cross-sectional survey from the USA involving 54 female patients aged 18 years and older with CHD also reported rates of disease severity, although in this sample, almost half of the participants reported severe disease [29]. A study from the USA involving 83 women aged 19 or older with CHD noted that 29% of participants were classified as having class 3 and 11% class 4 disease severity [25].

Finally, one study from the UK documented CVD severity among two groups of participants surveyed three years apart. In Group 1, using the mWHO classification, 14% of the participants were in stage II–III, and 14% in stage III. In Group 2, 31% were stage II–III and 14% were stage III. In total, 3% of the women were classified as WHO IV, in which pregnancy would be contraindicated [32]. In another study, 78% of participants had documentation regarding significant increased risk for an adverse pregnancy outcome. Interestingly, less than half of these women were able to self-report the risk correctly. Of all patients surveyed, 72% did not know that having CVD placed them at increased risk for having a child with CVD [29].

### Contraceptive counselling for women with CVD

**Fig. 2:**
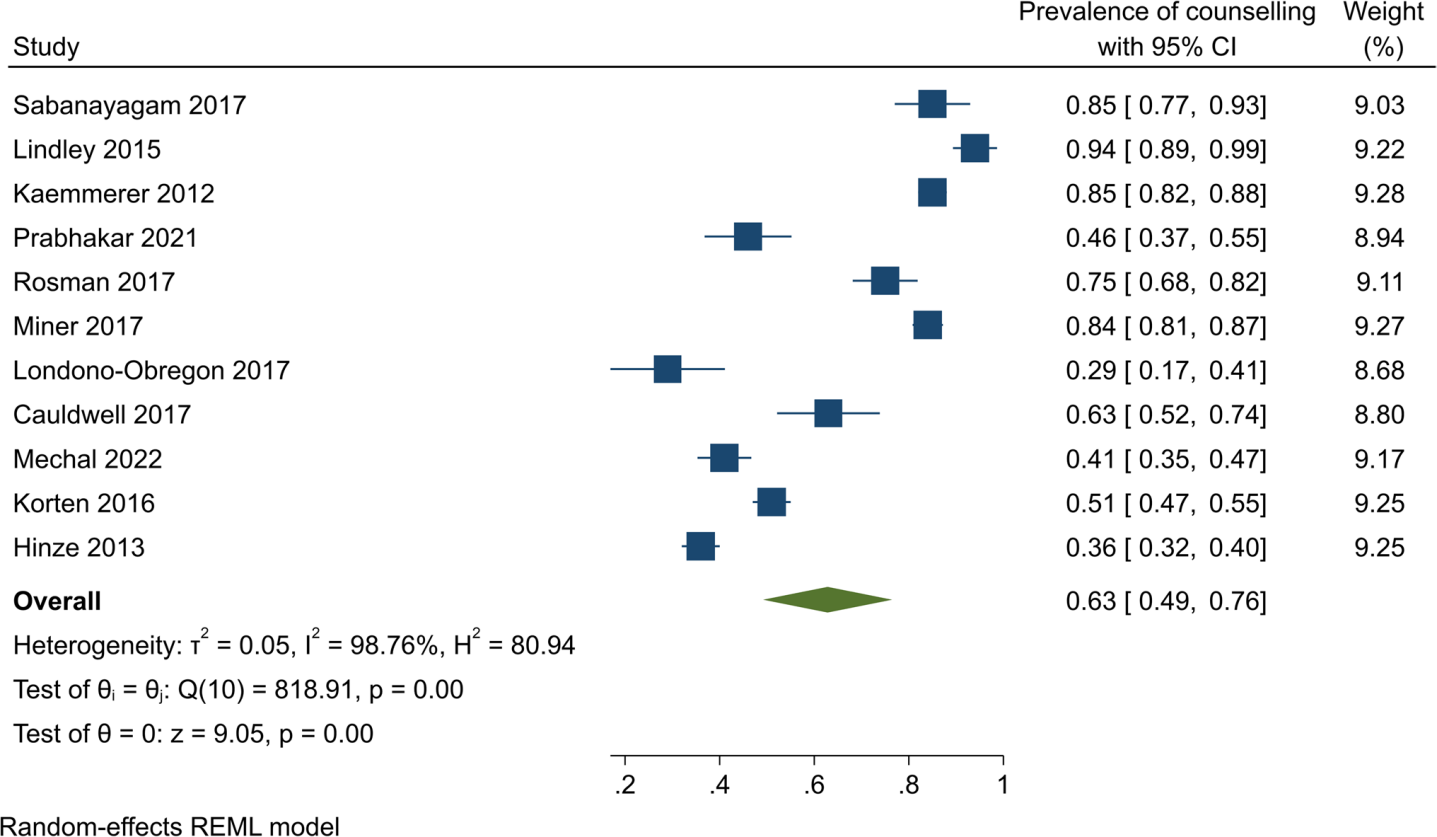
Contraceptive counselling among women with cardiovascular diseases

The pooled prevalence of contraceptive counselling was assessed using 11 studies. From these, the pooled prevalence was estimated at 63% (95% CI, 49–76%); however, there was significant evidence of heterogeneity (*Q* (10) = 818.19, *ɽ*2 = 0.05, *I*^2^ = 98.76%, *p* < 0.001) (Fig. 2).

**Fig. 3:**
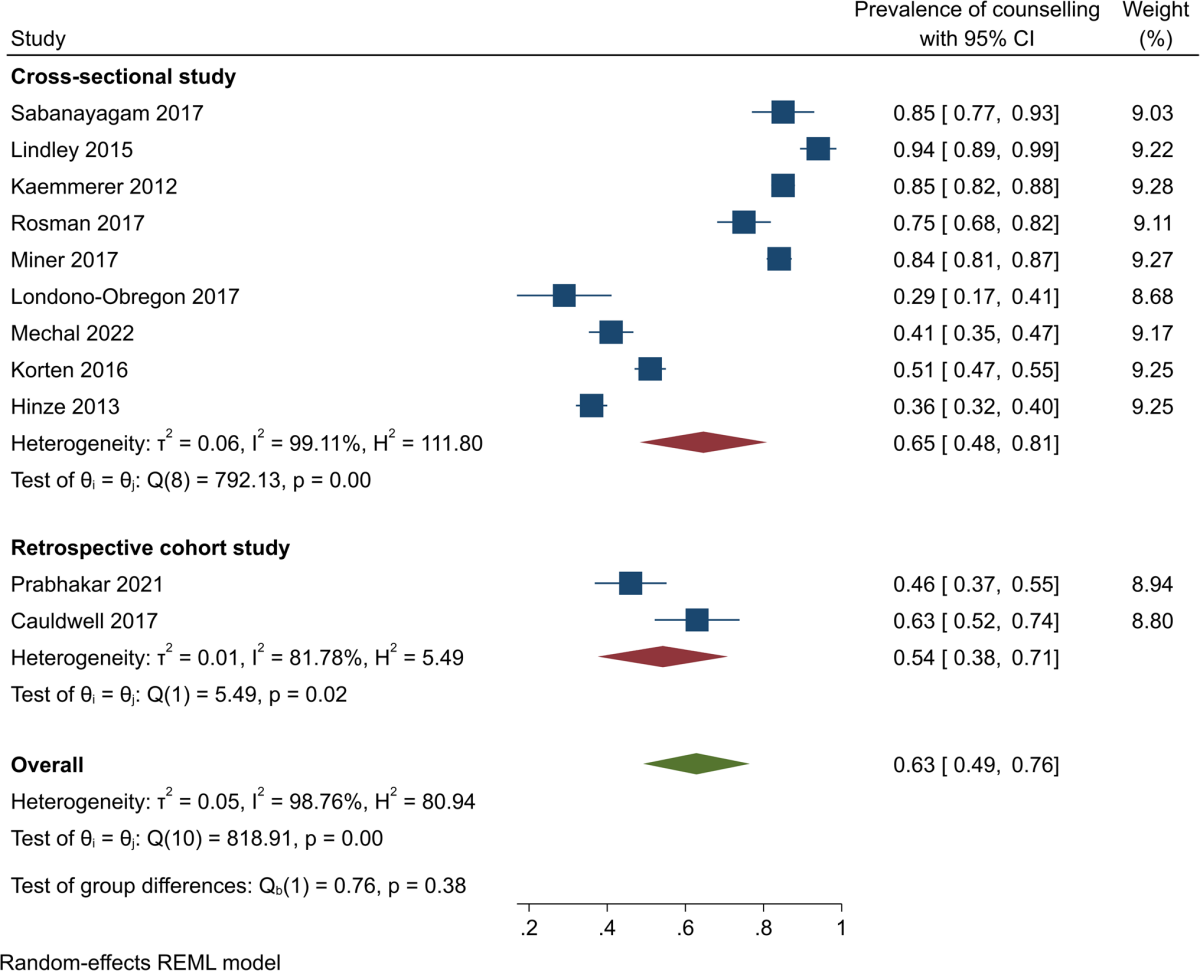
Contraceptive counselling by study design among women with cardiovascular diseases

**Fig. 4:**
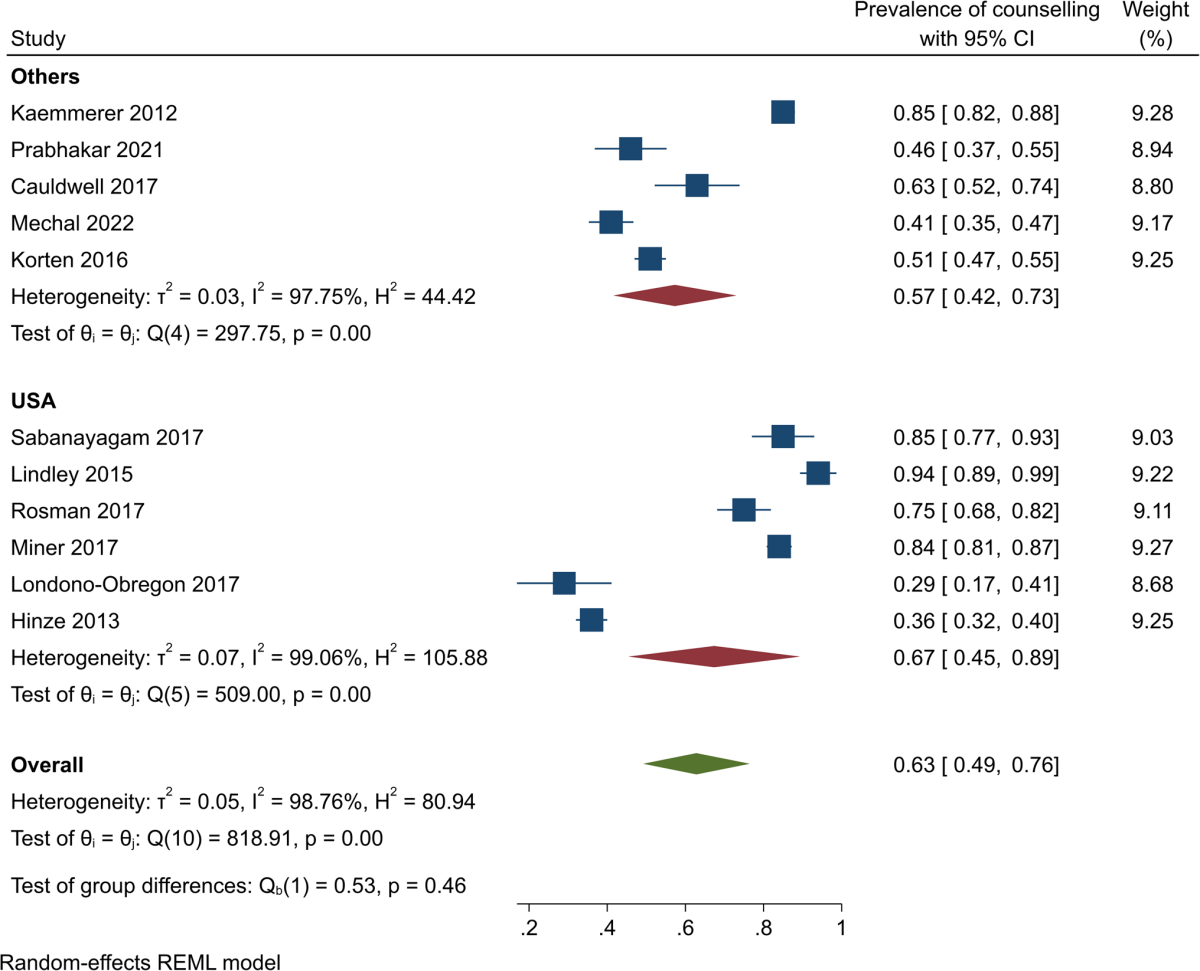
Contraceptive counselling by country among women with cardiovascular diseases

**Fig. 5:**
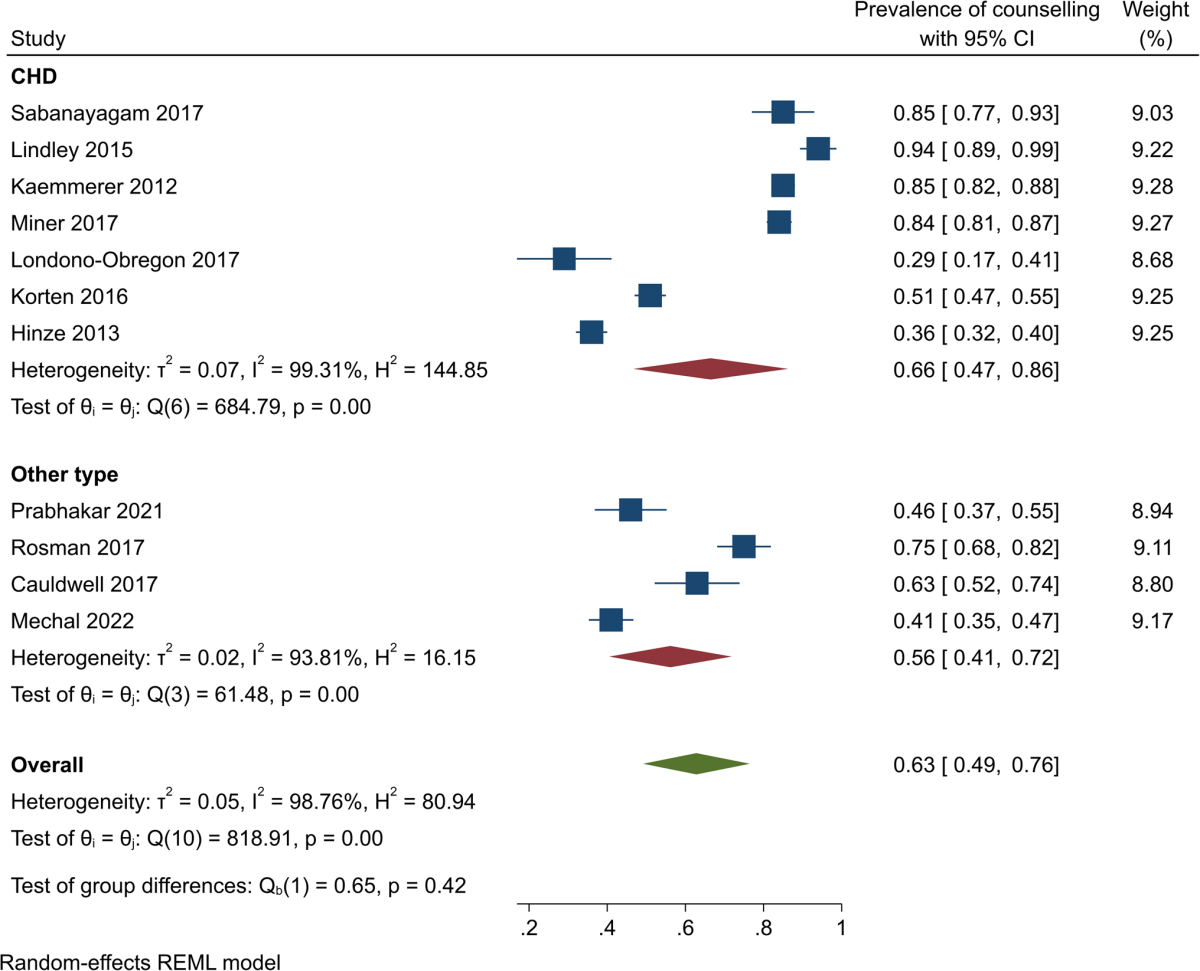
Contraceptive counselling by cardiovascular disease type

**Fig. 6:**
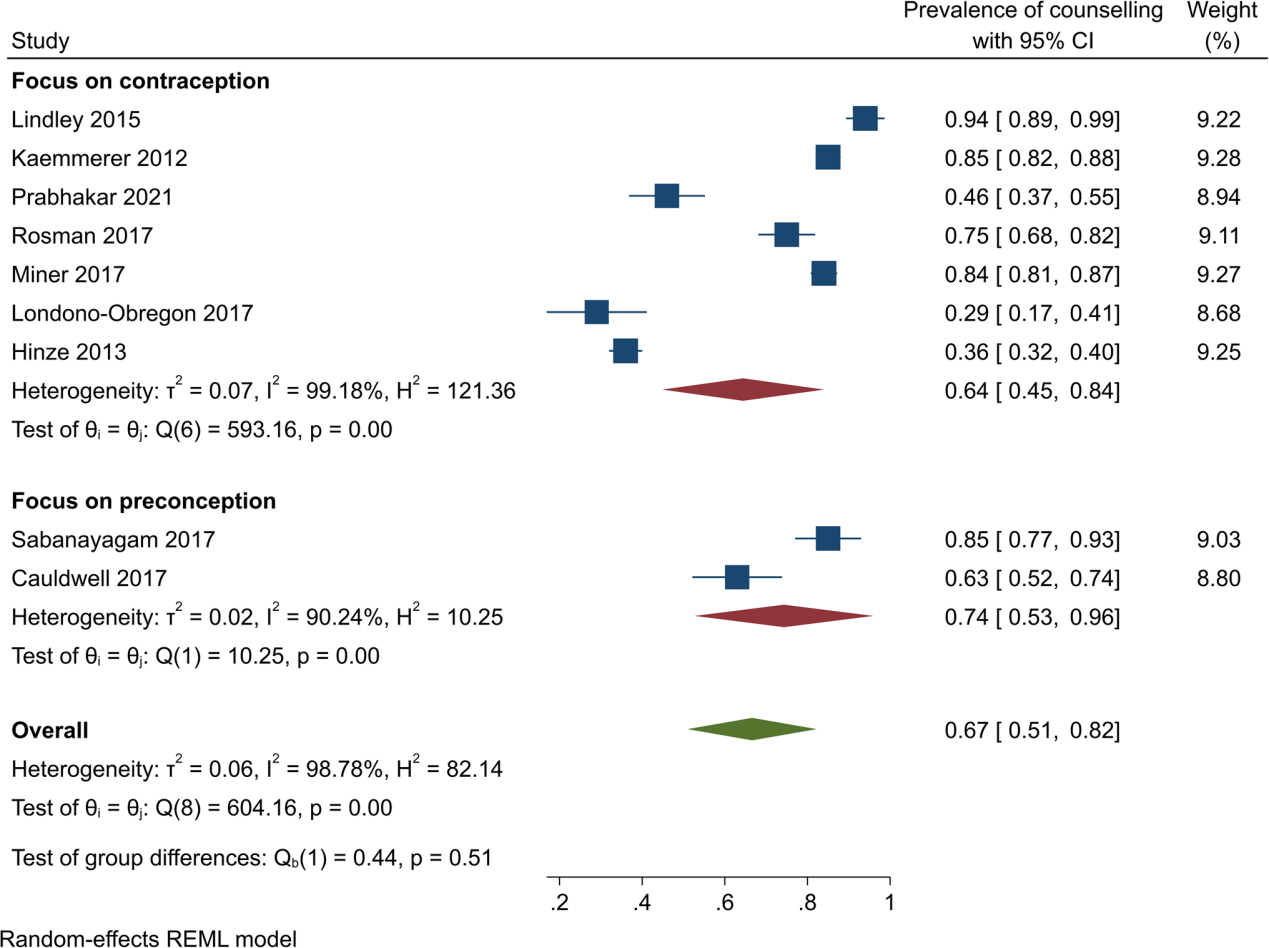
Contraceptive counselling by focus of the study

Subgroup analyses by study design showed that contraceptive counselling prevalence was 65% (95% CI, 48–81%) (*Q* (8) = 792.13, *I*^2^ = 99.11, *p* < 0.001) among cross-sectional studies and 54% (95% CI, 38–71%) (*Q* = 5.49, *I*^2^ = 81.78%, *p* < 0.001) among retrospective studies (Fig. 3). In US-based studies, the pooled contraceptive counselling prevalence was 67% (95% CI, 45–89%) (*Q* (5) = 509.00, *I*^2^ = 99.06, *p* < 0.001). In other countries, on pooling, 57% of participants received contraceptive counselling (95% CI, 42 to 73%) (*Q* (4) = 297.75, *I*^2^ = 97.75, *p* < 0.001) (Fig. 4). The pooled contraceptive counselling prevalence for CHD studies was 66% (95% CI, 47 to 86%) (*Q* (6) = 684.79, *I*^2^ = 99.31, *p* < 0.001) while in studies unrelated to CHD, the pooled contraceptive counselling prevalence was 56% (95% CI, 41–72%) (*Q* (3) = 61.48, *I*^2^ = 93.81, *p* < 0.001) (Fig. 5). In studies focusing on contraception provision, the contraceptive counselling prevalence was 64% (95% CI, 45–84%) (*Q* (6) = 593.16, *I*^2^ = 99.18, *p* < 0.001), while within the preconception focus studies, the contraceptive counselling prevalence was 74% (95% CI, 53–96% [*Q* (1) = 10.25, *I*^2^ = 90.24, *p* < 0.001]) (Fig. 6). Given the heterogeneity among these studies (*I*^2^ > 75%), a narrative review is provided below.

Furthemore, two studies found that about half of the participants did not receive contraceptive counselling inline with their specific heart conditions. A cross-sectional survey from the USA at a single tertiary adult CHD clinic showed 54% of women with CHD reported that a healthcare professional had never discussed their options for birth control with respect to their heart condition. Of the 38 participants who did report receiving contraceptive counselling that specifically addressed their heart condition, only 47% received such counselling prior to their first sexual intercourse [25]. A retrospective cohort study from the UK also highlighted that 63% of women with cardiac disease attended preconception counseling (PCC). Of the 14 that did not attend PCC, 5 referred to their service from another unit without PCC and 2 previously defaulted from follow-up [24].

Women with CVD however were more likely to receive counselling than women with alternative medical diagnoses. For instance, a retrospective comparative audit from the UK at a single tertiary maternity unit showed that nearly half of the women with an underlying cardiac condition had received counselling [32]. The same study compared two groups of participants. In Group 1 (conducted in 2015), this study highlighted those women with CHD were more likely to receive counselling compared to women with alternative diagnoses (64% vs. 29% (*p* < 0.01)). In Group 2 (conducted in 2018–2019), 46% received counselling (68% of the women with CHD vs. 15% of women with an alternative diagnosis (*p* < 0.001)) [32].

Four studies reported contraceptive counselling in relation to WHO’s risk of pregnancy classification (see Supplementary Table 3 for definition). A study among 88 women with CVD showed that women in higher pregnancy WHO classes were more likely to have received counselling than those in lower WHO classes (*P* < 0.002) [25]. In a UK retrospective study, in Group 1 (conducted in 2015), 69% of participants classified as high-risk received contraceptive counselling compared with 35% who were deemed low-risk (*p* < 0.05). In Group 2 (conducted in 2018–2019), 69% of the participants classified as high-risk (WHO II–III, III, or IV) received counselling compared with nine (26%) at low risk (*p* < 0.0001) [32]. Another study highlighted that women with complex CHD (according to the 2008 American College of Cardiology Guidelines for the Management of Adults with Congenital Heart Disease) were more likely to receive contraceptive counselling compared to those with less complex CHD (56.0% vs. 45%, *p* = 0.036) [31].

Furthermore, two studies documented a knowledge gap between actual and self-perceived disease severity among women with CHD. For example, a cross-sectional study from the USA [28], which applied the WHO Pregnancy Risk Classification to assess pregnancy complication risk, reported that only 51% of the study population accurately assessed their cardiovascular pregnancy risk score, with 22% underestimating and 27% overestimating the risk score [28]. In a cross-sectional study conducted across multiple centres in three countries (Germany, Hungary, and Japan), 45.7% (*n* = 290) of participants considered themselves to be inadequately informed, while 30.6% (*n* = 194) felt moderately informed, and less than one-fifth (*n* = 113) rated their level of information as very high [27].

Finally, there was a gap in knowledge regarding contraceptive choices, with many not having conversations with providers. A cross-sectional survey (*n* = 54) in an outpatient clinic of a large paediatric hospital in the USA indicated that less than half (46%) identified their contraceptive options correctly for their health conditions [29].

### Sources of contraception information

Eight studies identified that for women of reproductive age with CVD, their main sources of information were often obstetricians and gynaecologists, healthcare teams, and cardiologists [26–31, 33, 34]. Another study supported the findings that gynaecologists were the primary source of information. Only 43% indicated receiving counselling from their ACHD provider (physician, nurse practitioner, or physician assistant), 55% from gynaecologists, and 11% from primary care physicians [31]. A cross-sectional study from the USA reported that among participants who reported discussing contraception with a healthcare provider, 92.9% were with an obstetrician-gynaecologist, and less than 50% were with their cardiologist [28].

Two studies from the USA and the UK also showed that cardiologists were the primary source of heart-specific counselling for contraception among CHD patients, with some patients also receiving counselling from primary care physicians and obstetricians/gynaecologists [24, 25]. A cross-sectional survey from the USA at a single tertiary adult CHD clinic showed that a paediatric cardiologist was involved with 42% for their first contraceptive counselling and 13% for their most recent counselling. A cardiac specialist (paediatric cardiologist, adult cardiologist, or cardiothoracic surgeon) provided contraceptive counselling at least once for 57%, and only 25% of all study participants received heart-specific contraceptive counselling from a cardiac specialist. Others reported that heart-specific counselling first came from a primary care physician (26%) or an obstetrician/gynaecologist (24%). Regarding the most recent counselling, 29% received counselling from a primary care physician and 24% from an obstetrician/gynaecologist [25]. A UK retrospective cohort study also showed that combined ACHD cardiologist and obstetrician, as well as a follow-up from the cardiology team, were the source of contraceptive counselling [24]. Participants reported receiving counselling that did not include a discussion of their heart condition most frequently from a family medicine physician (73%) or an obstetrician/gynaecologist (55%) [25].

Finally, three studies from the USA and North America reported that ACHD providers and healthcare teams provided contraceptive counselling for more than a quarter of the participants. A cross-sectional survey in North America showed that 84% of ACHD reported receiving contraceptive counselling, but only 43% reported receiving counselling from an ACHD provider [31]. A USA cross-sectional study from registry also showed that the healthcare team provided information regarding the potential risks of subsequent pregnancies to 75.1% of patients [33]. While treating physicians were the primary source of contraceptive information, younger participants (aged 18–34 years) also turned to friends and the internet for information, with less than half discussing contraception with their cardiologist [26].

### Uptake of contraception among women with CVD

**Fig. 7:**
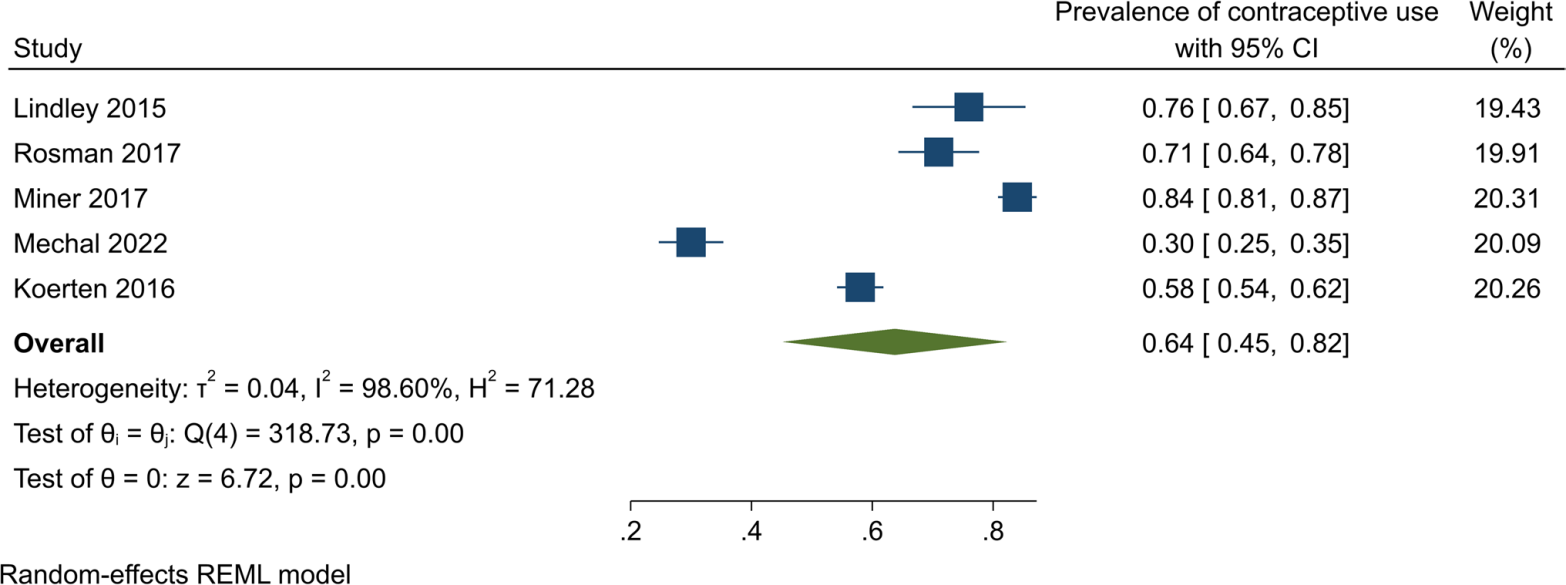
Contraceptive use among women with cardiovascular disorders

Among eleven studies included in this review, five assessed the prevalence of contraceptive uptake among women with CVD. The pooled contraceptive prevalence was 64% (95% CI, 45–82%); however, significant evidence of heterogeneity was identified (*Q* (4) = 318.73, *r*2 < 0.01, *I*^2^ = 98.06%, *p* < 0.001) (Fig. 7).

We found 64% of women with CVD were using some form of contraception. A cross-sectional study from the USA reported that approximately 76% of sexually active women with CHD were using contraceptives [28]. Only just over one-third of participants were using very effective contraceptive methods, with 11% using LARC methods (failure rate < 1% per year) [28]. In another US cross-sectional study, the commonly used LARC methods were tubal ligation (24.3%) and IUDs (16.4%) [33].

Some women were using less effective methods as well as methods where the risks of using the method outweighed the benefits. A cross-sectional study from the USA reported that only 24% of sexually active women with CHD were using effective methods (failure rate 6–12% a year), and 16% were using less-effective methods (failure rate 18–24% per year). This study found that 10% of participants with absolute or relative contraindications to combined hormonal methods were using them, with 53% reporting a history of use [28]. Three more studies reported many women with CVD, particularly those with CHD, were using less effective contraception (e.g., condoms) and often irregularly. A cross-sectional study from the USA showed that 15% of participants reported using contraception at least half or most of the time. The most common less effective method was condoms (22.0%) [33]. Another study however showed that women with CVD use a wide range of methods, with the most common methods including barrier methods, oral contraception, and progesterone-only pills [31]. A multi-country cross-sectional study found that condoms were the most commonly used method of contraception (38%), followed by oral contraceptives (30%) and withdrawal (11%) [27] (see Supplementary Fig. 1).

Finally, women with heart problems reported contraceptive choices that were inappropriate for their condition when there was a lack of heart-specific counselling. In a study of a single tertiary adult CVD clinic in the USA, 18% of women were found to have used a method where the risks might outweigh the benefits (contraindicated contraception). Most participants (63%) were not aware of specific methods not recommended due to their heart condition and medication use. Eight participants used combined hormonal contraception (CHC) class 4, and an additional 8 used CHC class 3, for a total of 16 participants deemed being at high risk for adverse events occurring secondary to the use of CHC [25]. Another Ethiopian hospital–based study found that women who had not received contraceptive counselling were more likely to have an unmet need for contraception compared to those who received counselling (AOR 6.7, CI 1.8–24.7) [30].

## Discussion

This systematic review and meta-analysis comprehensively examined contraceptive counselling and contraceptive uptake among women with CVD. We found about two-thirds of women with CVD received contraceptive counselling although this varied widely across individual studies. For women with CVD, there were large gaps in knowledge around the suitability of specific contraceptive methods for their specific condition. This is important given women with CVD had varying levels of risk classification for pregnancy, with many of them advised against pregnancy due to the type and severity of their medical condition.

While contraceptive counselling among women with CVD appears relatively high, this review identified that gaps still exist for these women in terms of contraceptive counselling, uptake of highly effective contraception, and knowledge regarding contraception suitability for their specific condition. One-third of women with CVD still miss out on contraceptive counselling, with counselling prioritised for those with severe disease. It is important to provide long-term comprehensive contraceptive care to all women with CVD, which not only safeguards their reproductive health but also plays a vital role in the overall management of their cardiovascular health. This review also reported that some women with CVD lack comprehensive knowledge about their condition and the appropriate contraceptive strategies tailored to their unique needs. Our review noted that contraceptive counselling was higher among women with CVD than other medical conditions [32]. While women with CVD receive more counselling, it is still haphazard with it based on disease severity. There is also the issue of uptake of appropriate contraception still being low despite this, so there are systematic and health provider issues that need to be addressed across the board for women with chronic disease.

When counselling women with CVD about contraception, it is important to consider that some women may require non-hormonal contraception [37]. Some hormonal contraceptives, notably combined oral contraceptive pills, carry a risk of increased blood pressure and blood clots [19, 20]. For example, women with a history of deep vein thrombosis or pulmonary embolism are generally not recommended to use combined oral contraceptive pills. On the other hand, women with well-controlled hypertension may be eligible for a lower-dose hormonal option, although this decision should be made following a thorough assessment of their health and must involve regular monitoring to ensure that blood pressure remains within safe limits. Progestogen-only contraceptives are generally considered safe for women with CVD and may be preferable for those unable to use combined oral contraceptive pills. Barrier methods (like condoms) and fertility awareness–based methods are considered safe but have very low efficacy [38, 39]. LARCs are a popular (the prevalence of LARC among women with chronic diseases was 23%) choice for women with CVD [40] because they are effective and safe. Comprehensive counselling across the disease course is therefore crucial, covering contraceptive choice, prevention of unintended pregnancy, planning pregnancy for times of better health, and switching of medication [14, 28, 40]. For example, it is crucial to understand contraceptive continuation or switching due to unwanted side effects, or perceived impact on future fertility, and how women prioritise different needs over time. Importantly, shared decision-making is a practical approach to healthcare that women at the centre of their care [41]. Women with CVD can make well-informed health and contraception decisions with a combination of healthcare expertise and online resources, as this approach may not only improve outcomes but also foster client-centred care. It may be important to conduct implementation research to determine the factors that predict successful contraceptive counselling.

Our review also highlighted that only 64% of women with CVD used some form of contraception. This finding is lower than the magnitude of contraceptive use at the time of their last vaginal sex for women with chronic disease in Australia (85.5%) [40]. However, despite overall higher use of contraception, this Australian longitudinal study highlighted that women with cardiac disease were twice as likely to use low efficacy contraception than women without chronic disease (using the pill) [40]. It is important to highlight that most studies included in this review were cross-sectional, and this Australian study is one of the few longitudinal studies specifically focused on chronic diseases. In our review, in individual studies, we also found that women with CVD were using less effective contraceptive methods (such as barrier methods), and moderately effective methods (such as combined hormonal contraceptives) [14]. We found that use of methods not recommended as the risks may outweigh the benefits of its use often occurs when counselling specific to heart conditions is missing. This often places women at risk of unintended pregnancies, leading to sometimes life-threatening situations for mothers and their babies [28], which has been observed among women with other medical conditions [15]. The variability in contraceptive prevalence and methods across studies suggests the need for a more comprehensive understanding of patient-related barriers. Many women may face barriers when it comes to choosing LARC, including concerns about possible side effects and misunderstandings about the methods [42]. Moreover, cultural or personal beliefs may also impact the decision-making process, as some women may have religious or cultural objections to certain forms of LARC [43]. So, understanding client-related barriers to their uptake is required. Importantly, education, counselling, and woman-centred and culturally appropriate care can help improve acceptance of LARC options [14].

Women with CVD often receive contraceptive counselling from multiple clinicians they encounter, and this situation mirrors findings in other health conditions, highlighting a need for coordination and role definition. Guidelines and policies for contraceptive counselling are crucial, given the increased number of reproductive-aged women with CVD [4]. Women with CVD desire disease-specific contraceptive information from their cardiologists and gynaecologists and importantly coordinated care among healthcare providers. However, healthcare providers are the gatekeepers of contraceptive knowledge, particularly regarding health conditions [44]. Guidelines and policies for contraceptive counselling among women with CVD can support contraceptive services and reduce unintended and adverse pregnancy outcomes. It is crucial to receive timely contraceptive counselling that identifies and facilitates access to contraceptive methods which not only consider heart conditions but also meet women's reproductive goals. Yet, our findings highlighted the lack of clear responsibility and communication issues can contribute to inadequate counselling [26–31, 33, 34]. Among studies that documented sources of contraceptive information, in our review, cardiologists and gynaecologists were the primary sources [28, 34] though younger participants tend to seek information from friends and the internet [26]. Addressing these gaps is crucial because many young women with CVD might be prescribed drugs that can cause birth defects [16, 45] if pregnancy occurs during this period. Younger women with CVD should also have access to safe and effective contraception to reduce risks associated with pregnancy [26]. Prioritising these conversations with a multidisciplinary team, including gynaecologists, cardiologists, and primary care providers, is essential although further research is also needed to determine the best way to incorporate contraceptive counselling into clinical practice [46, 47].

The strength of this review is a comprehensive search was conducted to find the best evidence available. To ensure the reliability of our review, we evaluated the characteristics of the included studies, including design, sample size, geographic location, and publication year. The limitations of the studies include factors such as a small sample size and the language, with only English language articles included. Heterogeneity of the studies was also noted, as different groups of participants were included for the pooling of the magnitude of contraceptive counselling and uptake of contraception. We also pooled the findings of different study designs, such as cross-sectional and retrospective studies, which may have resulted in less precise estimates. None of the studies provided information on the extended advantages and impacts of contraceptive counselling and use in women with CVD longitudinally. Due to the fewer number of studies, we could not conduct subgroup analyses for contraceptives among women with CVD across important factors, such as women’s age and education. Despite these limitations, this study provides a comprehensive overview of the recent findings on contraceptive counselling and uptake of contraception among women with CVD and will be valuable input for supporting the development of effective contraceptive intervention programs.

## Conclusion

This review underscores the lack of high-quality research on contraceptive counselling in the context of CVD. Contraceptive uptake among women with CVD is suboptimal, and there is a need for targeted contraceptive counselling to improve contraceptive uptake given a report of less effective contraceptive use. The disparities in contraceptive counselling prevalence across studies may indicate the need for standardized and comprehensive counselling interventions. Further implementation research may be needed to identify predictors of effective contraceptive counselling. Healthcare providers should also counsel contraceptive options for women with CVD for safer and most effective contraceptive methods (such as LARC being the most effective) in line with their reproductive goals and preferences. Identifying barriers to contraceptive counselling in relation to CVD types and risk stratification highlights the need for targeted studies to address some misconceptions, particularly those around the use of LARC. The team responsible for CVD care should also focus on providing clear advice to high-risk women about the safety and effectiveness of contraceptives to support women with CVD plan future pregnancies.

### Supplementary Information

Below is the link to the electronic supplementary material.

Supplementary file1 (DOCX 697 KB)

## Data Availability

Not applicable.

## Abbreviations

*ACHD*: Adult Congenital Heart Disease
*ACC*: American College of Cardiology
*CVD*: Cardiovascular disease
*CHC*: Combined hormonal contraception
*CHD*: Congenital health diseases
*IUDs*: Intrauterine devices
*JBI*: Joanna Briggs Institute
*LARCs*: Long-acting reversible contraception
*mWHO*: Modified World Health Organization
*PCC*: Preconception counselling
*PRISMA*: Preferred Reporting Items for Systematic Reviews and Meta-Analyses
