# Supplementary material for: Contraceptive counselling and uptake of contraception among women with cardiovascular diseases: a systematic review and meta-analysis
Source: Clin Res Cardiol. 2024 Jul 10;113(8):1151–70. doi: 10.1007/s00392-024-02472-9 (PMC11269356; doi:10.1007/s00392-024-02472-9)
Supplement: Supplementary file 1 — Supplementary file1 (DOCX 697 KB) [file 392_2024_2472_MOESM1_ESM.docx]

# Supplementary File 1: Search strategies

## Medline

Database(s): Ovid MEDLINE(R) and Epub Ahead of Print, In-Process, In-Data-Review & Other Non-Indexed Citations and Daily 1946 to May 01, 2023
Search Strategy:

| **#** | **Searches** | **Results** |
| --- | --- | --- |
| 1 | Cardi* Diseases.ti,ab. | 74422 |
| 2 | heart failure.ti,ab. | 203239 |
| 3 | congenital heart disease*.ti,ab. | 32736 |
| 4 | ((heart or cardiac or myocardial) adj2 (failure or infarct$ or attack$)).ti,ab. | 415651 |
| 5 | cardiac failure.ti,ab. | 12704 |
| 6 | myocardial infarction.ti,ab. | 204131 |
| 7 | heart disease.mp. or Heart Diseases/ | 263473 |
| 8 | arrhythmi*.ti,ab. | 104439 |
| 9 | tachycardi*.ti,ab. | 67427 |
| 10 | coronary*.ti,ab. | 440239 |
| 11 | pericard*.ti,ab. | 47627 |
| 12 | 1 or 2 or 3 or 4 or 5 or 6 or 7 or 8 or 9 or 10 or 11 | 1094742 |
| 13 | (counsel?ing or selection tool*).ti,ab. | 114339 |
| 14 | (education* adj (strateg* or program* or intervention*)).ti,ab. | 69875 |
| 15 | (decision* adj (tool* or aid*)).ti,ab. | 5411 |
| 16 | (quality adj4 (family planning or contracepti*)).ti,ab. | 860 |
| 17 | (patient adj3 (provider interaction* or provider communication*)).ti,ab. | 2217 |
| 18 | (client adj3 (provider interaction* or provider communication*)).ti,ab. | 119 |
| 19 | ((family planning or contracept*) adj4 (intervention* or implement* or therap*)).ti,ab. | 3466 |
| 20 | 13 or 14 or 15 or 16 or 17 or 18 or 19 | 192925 |
| 21 | (birth control or fertility regulation or contracepti* or family planning or birth control or depo?-medroxyprogesterone or depo? Medroxyprogesterone or Depo-Provera or IU?D or IUD or IUDS or IUS or intra?uterine device* or intra?uterine system* or implant* or implanon or jadelle or norplant*).ti,ab. | 563453 |
| 22 | Contraception/ or Contraceptive Agents/ or exp Contraceptive Agents, Female/ or Contraception Behavior/ or Contraceptive Devices/ or exp Contraceptive Devices, Female/ or Family Planning Services/ or *LONG-ACTING REVERSIBLE CONTRACEPTION/ | 116357 |
| 23 | (("birth control" or contracepti* or "family planning" or "fertility regulation") adj4 (continu* or discontinu* or uptake or initiat* or switch* or satisf* or using or use*)).ti,ab. | 34113 |
| 24 | 21 or 22 or 23 | 615435 |
| 25 | 20 and 24 | 13381 |
| 26 | (("birth control" or contracepti* or "family planning" or "fertility regulation") adj4 (counsel* or decision-making or "decision support" or "decision aid*" or "decision tool" or "decision tools" or educat* or "informed choice*" or select* or shared-decision* or "tiered-effectiveness")).ti,ab. | 6381 |
| 27 | (pre-concept* or preconcept*).ti,ab. | 8082 |
| 28 | Preconception Care/ | 2674 |
| 29 | 26 or 27 or 28 | 15541 |
| 30 | 25 or 28 | 15924 |
| 31 | 12 and 30 | 447 |
| 32 | limit 31 to (english language and humans and yr="2010 -Current") | 210 |

## EMBASE

Database(s): Embase 1947 to present

Search Strategy:

| **#** | **Searches** | **Results** |
| --- | --- | --- |
| 1 | Cardi* Diseases.ti,ab. | 116312 |
| 2 | heart failure.ti,ab. | 372457 |
| 3 | congenital heart disease*.ti,ab. | 55933 |
| 4 | ((heart or cardiac or myocardial) adj2 (failure or infarct$ or attack$)).ti,ab. | 703490 |
| 5 | cardiac failure.ti,ab. | 21994 |
| 6 | myocardial infarction.ti,ab. | 331767 |
| 7 | heart disease.mp. or Heart Diseases/ | 519712 |
| 8 | arrhythmi*.ti,ab. | 175328 |
| 9 | tachycardi*.ti,ab. | 120679 |
| 10 | coronary*.ti,ab. | 692094 |
| 11 | pericard*.ti,ab. | 78221 |
| 12 | 1 or 2 or 3 or 4 or 5 or 6 or 7 or 8 or 9 or 10 or 11 | 1814614 |
| 13 | (counsel?ing or selection tool*).ti,ab. | 179774 |
| 14 | (education* adj (strateg* or program* or intervention*)).ti,ab. | 101004 |
| 15 | (decision* adj (tool* or aid*)).ti,ab. | 8408 |
| 16 | (quality adj4 (family planning or contracepti*)).ti,ab. | 881 |
| 17 | (patient adj3 (provider interaction* or provider communication*)).ti,ab. | 3153 |
| 18 | (client adj3 (provider interaction* or provider communication*)).ti,ab. | 132 |
| 19 | ((family planning or contracept*) adj4 (intervention* or implement* or therap*)).ti,ab. | 4763 |
| 20 | 13 or 14 or 15 or 16 or 17 or 18 or 19 | 293064 |
| 21 | (birth control or fertility regulation or contracepti* or family planning or birth control or depo?-medroxyprogesterone or depo? Medroxyprogesterone or Depo-Provera or IU?D or IUD or IUDS or IUS or intra?uterine device* or intra?uterine system* or implant* or implanon or jadelle or norplant*).ti,ab. | 818048 |
| 22 | Contraception/ or Contraceptive Agents/ or exp Contraceptive Agents, Female/ or Contraception Behavior/ or Contraceptive Devices/ or exp Contraceptive Devices, Female/ or Family Planning Services/ or *LONG-ACTING REVERSIBLE CONTRACEPTION/ | 262791 |
| 23 | (("birth control" or contracepti* or "family planning" or "fertility regulation") adj4 (continu* or discontinu* or uptake or initiat* or switch* or satisf* or using or use*)).ti,ab. | 42562 |
| 24 | 21 or 22 or 23 | 974401 |
| 25 | 20 and 24 | 19551 |
| 26 | (("birth control" or contracepti* or "family planning" or "fertility regulation") adj4 (counsel* or decision-making or "decision support" or "decision aid*" or "decision tool" or "decision tools" or educat* or "informed choice*" or select* or shared-decision* or "tiered-effectiveness")).ti,ab. | 7839 |
| 27 | (pre-concept* or preconcept*).ti,ab. | 13160 |
| 28 | Preconception Care/ | 3100 |
| 29 | 26 or 27 or 28 | 22334 |
| 30 | 25 or 28 | 22494 |
| 31 | 12 and 30 | 985 |
| 32 | limit 31 to (english language and humans and yr="2010 -Current") | 639 |

## CINAHL

| 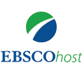 | Wednesday, April 26, 2023 11:27:37 AM |
| --- | --- |

| **#** | **Query** | **Limiters/Expanders** | **Last Run Via** | **Results** |
| --- | --- | --- | --- | --- |
| S1 | ( ( “Cardiovascular Disease” or “myocardial infarction” or “Congenital heart disease” or “heart failure” or “heart attack” or “cardiac failure” or “((heart or cardiac or myocardial) near/2 (failure or infarct or attack))” ) ) AND ( ( “preconception care” or “prepregnancy” or “pregestation” or “pregravid” or “family planning service” or “family planning counselling” or “contraception counselling” or “contraceptive counselling” or “reproductive health service” or “Fertility” or “reproductive health” or “sexual health” or “women's health” or “birth control” or “pre-pregnancy” or “pre-gestation” or “pre-gravid” or “pre-conception” or “before pregnancy” ) ) | Limiters - Published Date: 20100101-20230531; English Language; Exclude MEDLINE records; Human; Sex: Female  Expanders - Apply equivalent subjects  Search modes - Boolean/Phrase | Interface - EBSCOhost Research Databases  Search Screen - Advanced Search  Database - CINAHL Complete | 346 |

## MIDRIS

Database(s): Maternity & Infant Care Database (MIDIRS) 1971 to April 18, 2023

Search Strategy:

| **#** | **Searches** | **Results** |
| --- | --- | --- |
| 1 | Cardi* Diseases.ti,ab. | 253 |
| 2 | heart failure.ti,ab. | 502 |
| 3 | congenital heart disease*.ti,ab. | 1625 |
| 4 | ((heart or cardiac or myocardial) adj2 (failure or infarct$ or attack$)).ti,ab. | 869 |
| 5 | cardiac failure.ti,ab. | 167 |
| 6 | myocardial infarction.ti,ab. | 188 |
| 7 | (heart disease or Heart Diseases).ti,ab. | 2402 |
| 8 | arrhythmi*.ti,ab. | 431 |
| 9 | tachycardi*.ti,ab. | 549 |
| 10 | coronary*.ti,ab. | 467 |
| 11 | pericard*.ti,ab. | 148 |
| 12 | 1 or 2 or 3 or 4 or 5 or 6 or 7 or 8 or 9 or 10 or 11 | 4375 |
| 13 | (counsel?ing or selection tool*).ti,ab. | 8187 |
| 14 | (education* adj (strateg* or program* or intervention*)).ti,ab. | 2413 |
| 15 | (decision* adj (tool* or aid*)).ti,ab. | 155 |
| 16 | (quality adj4 (family planning or contracepti*)).ti,ab. | 99 |
| 17 | (patient adj3 (provider interaction* or provider communication*)).ti,ab. | 61 |
| 18 | (client adj3 (provider interaction* or provider communication*)).ti,ab. | 20 |
| 19 | ((family planning or contracept*) adj4 (intervention* or implement* or therap*)).ti,ab. | 181 |
| 20 | 13 or 14 or 15 or 16 or 17 or 18 or 19 | 10850 |
| 21 | (birth control or fertility regulation or contracepti* or family planning or birth control or depo?-medroxyprogesterone or depo? Medroxyprogesterone or Depo-Provera or IU?D or IUD or IUDS or IUS or intra?uterine device* or intra?uterine system* or implant* or implanon or jadelle or norplant*).ti,ab. | 6973 |
| 22 | (Contraception or Contraceptive Agents or Contraceptive Agents or Contraception Behavior or Contraceptive Devices or Family Planning).ti,ab. | 4222 |
| 23 | (("birth control" or contracepti* or "family planning" or "fertility regulation") adj4 (continu* or discontinu* or uptake or initiat* or switch* or satisf* or using or use*)).ti,ab. | 2298 |
| 24 | 21 or 22 or 23 | 6973 |
| 25 | 20 and 24 | 1063 |
| 26 | (("birth control" or contracepti* or "family planning" or "fertility regulation") adj4 (counsel* or decision-making or "decision support" or "decision aid*" or "decision tool" or "decision tools" or educat* or "informed choice*" or select* or shared-decision* or "tiered-effectiveness")).ti,ab. | 710 |
| 27 | (pre-concept* or preconcept*).ti,ab. | 2567 |
| 28 | Preconception Care.ti,ab. | 414 |
| 29 | 26 or 27 or 28 | 3239 |
| 30 | 25 or 28 | 1455 |
| 31 | 12 and 30 | 19 |
| 32 | limit 31 to (english language and humans and yr="2010 -Current") [Limit not valid; records were retained] | 13 |

## PsycInfo (OVID)

Database(s): **APA PsycInfo**1806 to June Week 1 2023
Search Strategy:

| **#** | **Searches** | **Results** |
| --- | --- | --- |
| 1 | Cardi* Diseases.ti,ab. | 2989 |
| 2 | heart failure.ti,ab. | 4666 |
| 3 | congenital heart disease*.ti,ab. | 803 |
| 4 | ((heart or cardiac or myocardial) adj2 (failure or infarct$ or attack$)).ti,ab. | 10039 |
| 5 | cardiac failure.ti,ab. | 140 |
| 6 | myocardial infarction.ti,ab. | 4520 |
| 7 | heart disease.mp. or Heart Diseases/ | 11601 |
| 8 | arrhythmi*.ti,ab. | 3873 |
| 9 | tachycardi*.ti,ab. | 1705 |
| 10 | coronary*.ti,ab. | 11002 |
| 11 | pericard*.ti,ab. | 189 |
| 12 | 1 or 2 or 3 or 4 or 5 or 6 or 7 or 8 or 9 or 10 or 11 | 32574 |
| 13 | (counsel?ing or selection tool*).ti,ab. | 91078 |
| 14 | (education* adj (strateg* or program* or intervention*)).ti,ab. | 43870 |
| 15 | (decision* adj (tool* or aid*)).ti,ab. | 1836 |
| 16 | (quality adj4 (family planning or contracepti*)).ti,ab. | 121 |
| 17 | (patient adj3 (provider interaction* or provider communication*)).ti,ab. | 1109 |
| 18 | (client adj3 (provider interaction* or provider communication*)).ti,ab. | 47 |
| 19 | ((family planning or contracept*) adj4 (intervention* or implement* or therap*)).ti,ab. | 398 |
| 20 | 13 or 14 or 15 or 16 or 17 or 18 or 19 | 135980 |
| 21 | (birth control or fertility regulation or contracepti* or family planning or birth control or depo?-medroxyprogesterone or depo? Medroxyprogesterone or Depo-Provera or IU?D or IUD or IUDS or IUS or intra?uterine device* or intra?uterine system* or implant* or implanon or jadelle or norplant*).ti,ab. | 30814 |
| 22 | Contraception/ or Contraceptive Agents/ or exp Contraceptive Agents, Female/ or Contraception Behavior/ or Contraceptive Devices/ or exp Contraceptive Devices, Female/ or Family Planning Services/ or *LONG-ACTING REVERSIBLE CONTRACEPTION/ | 5022 |
| 23 | (("birth control" or contracepti* or "family planning" or "fertility regulation") adj4 (continu* or discontinu* or uptake or initiat* or switch* or satisf* or using or use*)).ti,ab. | 5713 |
| 24 | 21 or 22 or 23 | 31273 |
| 25 | 20 and 24 | 2176 |
| 26 | (("birth control" or contracepti* or "family planning" or "fertility regulation") adj4 (counsel* or decision-making or "decision support" or "decision aid*" or "decision tool" or "decision tools" or educat* or "informed choice*" or select* or shared-decision* or "tiered-effectiveness")).ti,ab. | 1322 |
| 27 | (pre-concept* or preconcept*).ti,ab. | 2518 |
| 28 | Preconception Care/ | 0 |
| 29 | 26 or 27 or 28 | 3817 |
| 30 | 25 or 28 | 2176 |
| 31 | 12 and 30 | 15 |
| 32 | limit 31 to (english language and humans and yr="2010 -Current") [Limit not valid in APA PsycInfo; records were retained] | 12 |

## Google Scholar

"Contraceptive counselling cardiovascular diseases" "uptake of contraception women"- 8 articles

# Supplementary Figure 1: Contraceptive methods as reported by primary studies


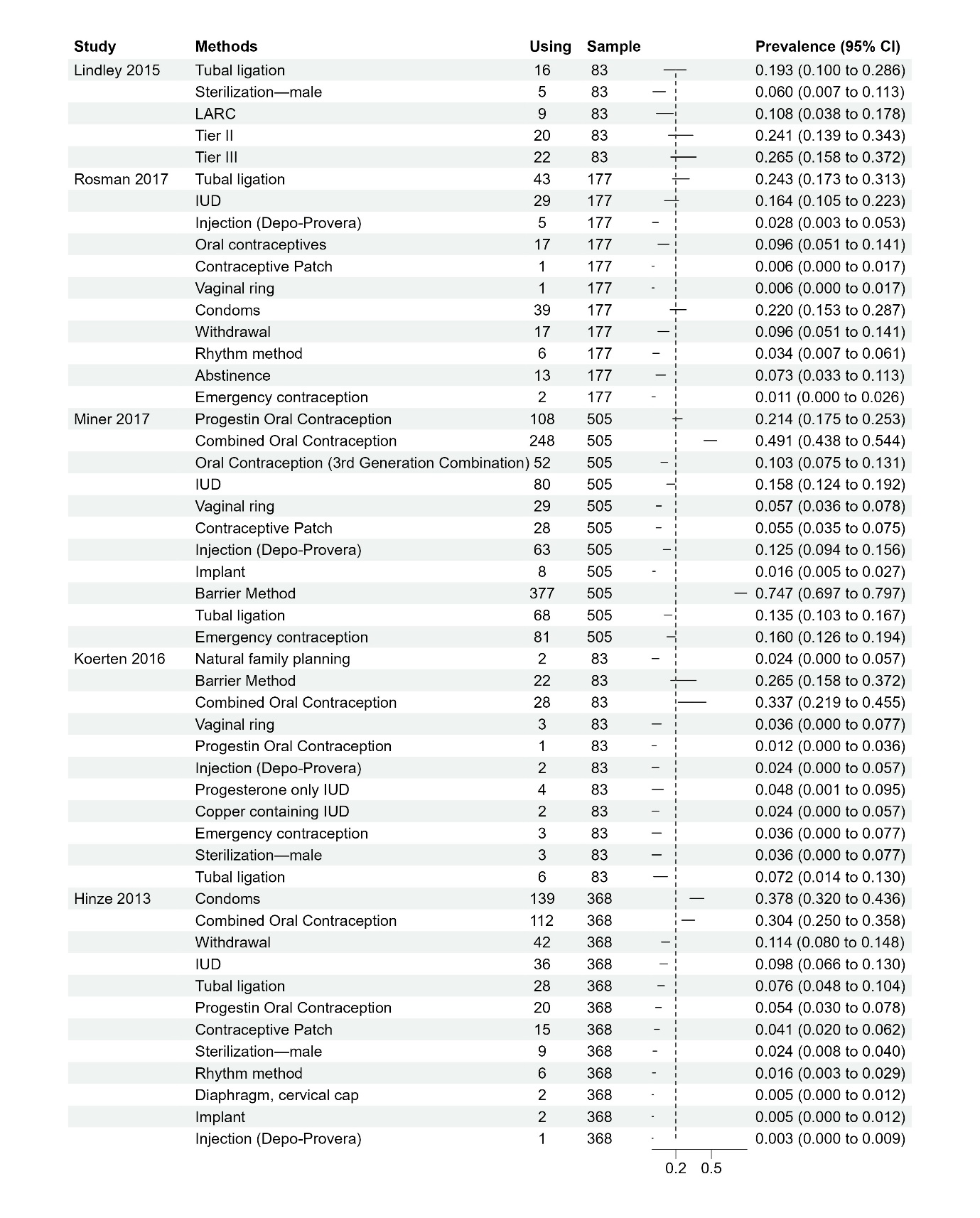


# Supplementary Table 1: Quality appraisal for included cross-sectional studies

| Author (year) | Item 1 | Item 2 | Item 3 | Item 4 | Item 5 | Item 6 | Item 7 | Item 8 | Overall Appraisal |
| --- | --- | --- | --- | --- | --- | --- | --- | --- | --- |
| Lindley 2015 (33) | Yes | Yes | Yes | Yes | No | No | Yes | Yes | 6 |
| Sabanayagam 2017 (27) | Yes | No | Yes | Yes | No | No | No | No | 3 |
| Rosman 2017 (28) | Yes | Yes | N/A | Yes | No | No | Yes | No | 4 |
| Kaemmerer 2012 (35) | Yes | Yes | Yes | Yes | Yes | Yes | Yes | Yes | 8 |
| Miner 2017 (29) | Yes | Yes | No | Yes | No | No | Yes | No | 4 |
| Londono-Obregon 2017 (30) | Yes | Yes | No | Yes | No | No | Yes | No | 4 |
| Hinze 2013 (34) | Yes | Yes | Yes | Yes | No | No | Yes | Yes | 6 |
| Mechal 2022 (26) | Yes | Yes | N/A | Yes | N/A | N/A | Yes | Yes | 5 |
| Koerten 2016 (32) | Yes | Yes | No | Yes | Yes | Yes | Yes | No | 6 |

Keys

1. Were the criteria for inclusion in the sample clearly defined?
2. Were the study subjects and the setting described in detail?
3. Was the exposure measured in a valid and reliable way?
4. Were objective, standard criteria used for measurement of the condition?
5. Were confounding factors identified?
6. Were strategies to deal with confounding factors stated?
7. Were the outcomes measured in a valid and reliable way?
8. Was appropriate statistical analysis used?

# Supplementary Table 2: Quality appraisal for included longitudinal studies

| Longitudinal studies | Item 1 | Item 2 | Item 3 | Item 4 | Item 5 | Item 6 | Item 7 | Item 8 | Item 9 | Item 10 | Item 11 | Overall Appraisal |
| --- | --- | --- | --- | --- | --- | --- | --- | --- | --- | --- | --- | --- |
| Prabhakar 2021 (25) | No | Yes | Yes | Yes | No | Yes | Yes | No | No | No | Yes | 6 |
| Cauldwell 2017 (31) | No | Yes | Yes | Yes | No | Yes | Yes | No | No | No | Yes | 6 |

Keys

1. Were the two groups similar and recruited from the same population?
2. Were the exposures measured similarly to assign people to both exposed and unexposed groups?
3. Was the exposure measured in a valid and reliable way?
4. Were confounding factors identified?
5. Were strategies to deal with confounding factors stated?
6. Were the groups/participants free of the outcome at the start of the study (or now of exposure)?
7. Were the outcomes measured in a valid and reliable way?
8. Was the follow up time reported and sufficient to be long enough for outcomes to occur?
9. Was follow up complete, and if not, were the reasons to loss to follow up described and explored?
10. Were strategies to address incomplete follow up utilised?
11. Was appropriate statistical analysis used?

# Supplementary Table 3: Categories and definition of WHO’s risk of pregnancy classification.

| WHO Category | Definition |
| --- | --- |
| Class 1 | The risk of maternal morbidity and mortality is not detectably higher than that of the general population. |
| Class 2 | Certain heart conditions carry a small increased risk of maternal mortality or morbidity |
| Class 3 | Specific heart conditions have a significant increased risk of maternal morbidity or mortality. Women with such conditions need expert joint cardiac and obstetric preconception counselling and care throughout the antenatal and peripartum period |
| Class 4 | Certain heart conditions carry an extremely high risk of maternal mortality or severe morbidity, and pregnancy is contraindicated. If pregnancy occurs, termination should be discussed, and if it continues, the care should be as for WHO 3. |
